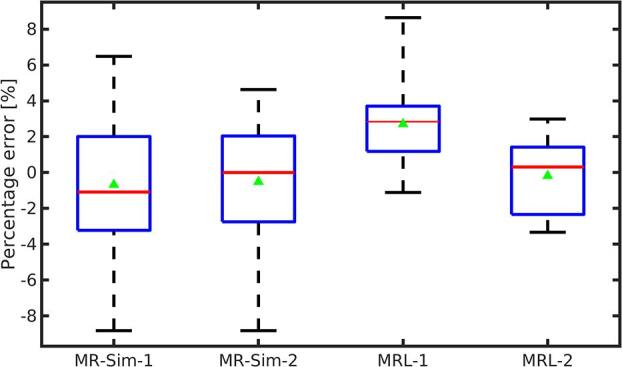# Feasibility of cardiac-synchronized quantitative T_1_ and T_2_ mapping on a hybrid 1.5 Tesla magnetic resonance imaging and linear accelerator system

**DOI:** 10.1016/j.phro.2022.02.017

**Published:** 2022-03-09

**Authors:** Osman Akdag, Stefano Mandija, Astrid L.H.M.W. van Lier, Pim T.S. Borman, Tim Schakel, Eveline Alberts, Oscar van der Heide, Rutger J. Hassink, Joost J.C. Verhoeff, Firdaus A.A. Mohamed Hoesein, Bas W. Raaymakers, Martin F. Fast

**Affiliations:** a Department of Radiotherapy, University Medical Center Utrecht, Heidelberglaan 100, 3584 CX Utrecht, The Netherlands; b Computational Imaging Group for MR Diagnostics and Therapy, Center for Image Sciences, University Medical Center Utrecht, Heidelberglaan 100, 3584 CX Utrecht, The Netherlands; c Philips Healthcare, Veenpluis 6 5684 PC Best, The Netherlands; d Department of Cardiology, University Medical Center Utrecht, Heidelberglaan 100, 3584 CX Utrecht, The Netherlands

**Keywords:** MR-linac, MRI, Quantitative cardiac MRI, MRI-guided radiotherapy, Cardiac MR-linac

## Abstract

**Background and Purpose:**

The heart is important in radiotherapy either as target or organ at risk. Quantitative T_1_ and T_2_ cardiac magnetic resonance imaging (qMRI) may aid in target definition for cardiac radioablation, and imaging biomarker for cardiotoxicity assessment. Hybrid MR-linac devices could facilitate daily cardiac qMRI of the heart in radiotherapy. The aim of this work was therefore to enable cardiac-synchronized T_1_ and T_2_ mapping on a 1.5 T MR-linac and test the reproducibility of these sequences on phantoms and *in vivo* between the MR-linac and a diagnostic 1.5 T MRI scanner.

**Materials and methods:**

Cardiac-synchronized MRI was performed on the MR-linac using a wireless peripheral pulse-oximeter unit. Diagnostically used T_1_ and T_2_ mapping sequences were acquired twice on the MR-linac and on a 1.5 T MR-simulator for a gel phantom and 5 healthy volunteers in breath-hold. Phantom T_1_ and T_2_ values were compared to gold-standard measurements and percentage errors (PE) were computed, where negative/positive PE indicate underestimations/overestimations. Manually selected regions-of-interest were used for *in vivo* intra/inter scanner evaluation.

**Results:**

Cardiac-synchronized T_1_ and T_2_ qMRI was enabled after successful hardware installation on the MR-linac. From the phantom experiments, the measured T_1_/T_2_ relaxation times had a maximum percentage error (PE) of −4.4%/−8.8% on the MR-simulator and a maximum PE of −3.2%/+8.6% on the MR-linac. Mean T_1_/T_2_ of the myocardium were 1012±34/51±2 ms on the MR-simulator and 1034±42/51±1 ms on the MR-linac.

**Conclusions:**

Accurate cardiac-synchronized T_1_ and T_2_ mapping is feasible on a 1.5 T MR-linac and might enable novel plan adaptation workflows and cardiotoxicity assessments.

## 1 Introduction

The heart is considered increasingly important in radiotherapy either as a target site or an organ at risk (OAR). Especially in lung and breast irradiations, it is important to avoid the heart as much as possible to prevent cardiotoxicity [1], [2]. More recently, non-invasive stereotactic arrhythmia radioablation (STAR) has emerged as salvage treatment option for patients with recurrent ventricular tachycardia (VT) [3], [4], [5]. VT is a severe cardiac arrhythmia disorder and a major risk factor for sudden cardiac death. Re-entrant circuits originating within the border zone surrounding a myocardial scar commonly cause VT [6], [7], [8]. During STAR, a single high-dose fraction (typically 1×25 Gy) is targeted at the VT substrate in the left ventricle. Cardiac radioablation was also performed to treat a patient with atrial fibrillation [9]. In an oncological setting, recent case reports describe the treatment of patients with cardiac sarcomas using MR-guided radiotherapy [10], [11].

Cardiac MRI (CMR) is a well established diagnostic imaging modality for the assessment of cardiac function and anatomy [12], but its application on MR-linac systems is not yet exploited. In particular, quantitative T_1_ and T_2_ CMR techniques may allow for cardiac tissue characterization without the need of contrast agent administration. Late gadolinium enhanced (LGE)-MRI is clinically used for diagnostic myocardial scar imaging [13], [14], but is currently not advisable for online MR-guided radiotherapy due to the unproven in vivo safety profile [15]. Omitting the necessity of a contrast agent would also diminish concerns when treating patients with contraindications for contrast agent administration [16].

Cardiac T_1_ and T_2_ mapping techniques on hybrid MR-linac devices could therefore facilitate novel plan adaptation workflows for STAR and cardiotoxicity assessments (without requiring additional scan sessions). For example, the increased fibrosis condition within a myocardial scar leads to higher T_1_ relaxation times and ensures differentiation between healthy and scarred cardiac tissue [17]. On the MR-linac, native T1 mapping could therefore become an option for myocardial scar imaging to guide STAR treatments. Native T_1_ mapping could also be indicative of radiation-induced tissue remodelling [18], [19]. Native T_2_ mapping can be applied to characterize the presence of edema, which is commonly a result of an, potentially radiation-induced, acute inflammatory reaction [20], [21], [22], [23], [24]. The aforementioned capabilities of quantitative T_1_ and T_2_ CMR methods could make these quantitative CMR methods front-runners for the guidance of VT-treatments and/or assessment of radiation-induced cardiotoxicity on the MR-linac systems.

Quantitative T_1_ and T_2_ MRI is widely applied in diagnostic CMR imaging protocols for cardiovascular patients [25]. However, in radiotherapy, the use of quantitative T_1_ and T_2_ MRI is novel and largely unexplored. First evidences of quantitative T_1_ and T_2_ MRI methods on the MR-linac were reported by Kooreman et al. [26]. In that study, the authors demonstrated the feasibility, accuracy and reproducibility of quantitative T_1_ and T_2_ mapping on different MR-linac systems. Crucially, their study did not include cardiac synchronisation, which is fundamental for cardiac imaging applications. A performance comparison with either a diagnostic MRI system or ground-truth T_1_ and T_2_ mapping sequences was also not part of their study.

The purpose of this study was to enable cardiac-synchronized CMR acquisitions on a 1.5 T MR-linac system for the first time and to demonstrate the feasibility of measuring cardiac-synchronized T_1_ and T_2_ maps on the 1.5 T MR-linac system for guidance and assessment of RT treatments in the thoracic region (e.g., STAR treatments). Furthermore, we aimed to quantify the intra/inter scanner reproducibility by acquiring the T_1_ and T_2_ maps in phantom gel samples and 5 healthy volunteers on an MR-simulator (i.e., a diagnostic MRI scanner with RT planning functionalities) and the MR-linac by using standard clinically used diagnostics T_1_ and T_2_ mapping sequences. Gold-standard T_1_ and T_2_ sequences were acquired on the phantom to attain ground-truth reference values.

## 2 Material and methods

### 2.1 Subjects and experimental setup

**Supplementary Fig. 1:**
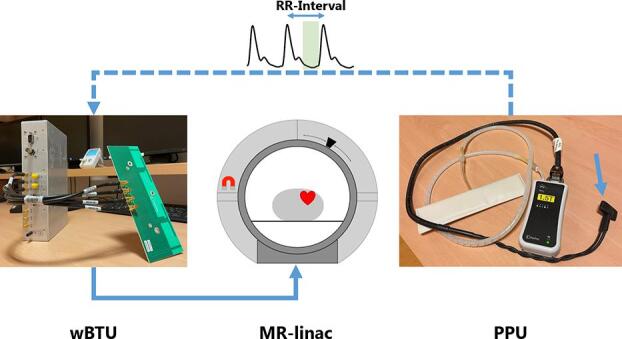


A wide-bore whole body 1.5 T Ingenia MRI scanner (Philips Healthcare, Best, The Netherlands) equipped with a 16-channel anterior and a 16-channel posterior coil array was used. For cardiac synchronization, a peripheral pulse-oximeter unit (PPU) was used. The Elekta Unity MR-linac contains a 1.5 T MRI system equipped with a 4-channel anterior and a 4-channel posterior coil array. Cardiac synchronization was enabled on the MR-linac in research mode. In collaboration with Philips Healthcare, a wireless basic triggering unit (wBTU) was installed in the treatment room and connected to the trigger input-line of the MRI-system to enable cardiac synchronization using the PPU (Supplementary Fig. 1). It has to be noted that by using a PPU an intrinsic delay of about 250 ms with respect to the heartbeat is introduced. However, this can be compensated by adjusting the acquisition delay accordingly.

The Eurospin TO5 phantom (Diagnostic Sonar, Livingston, Scotland) was used for robustness and reproducibility evaluations of the T_1_ and T_2_ mapping sequences. The phantom consisted of 16 gel samples with vendor-provided T_1_ relaxation times between 329 and 1603 (± 3%) ms and T_2_ relaxation times between 49 and 373 (± 3%) ms at 296 K at 1.5 T. By using a calibrated phantom, ground-truth reference values could be acquired in controlled settings using gold-standard T_1_ and T_2_ mapping sequences and could therefore be compared with the T_1_ and T_2_ values obtained with the clinically used sequences (see below in data collection for sequence details).

A total of five healthy volunteers (two female and three male, mean age ± SD  = 29.3 ± 5.3 years, mean BMI ± SD  = 21.7 ± 1.4, mean cardiac frequency ± SD  = 73 ± 15 bpm) were included in this study (study ID: NL59820.041.17) after obtaining written informed consent.

A balanced steady-state free precession (bSSFP) imaging sequence (TR/TE  = 2.7/1.4 ms, flip angle (FA) = 45°, field of view (FOV) = 350×294 mm^2^, voxel size = 2.7×3.6×8.0 mm^3^) was used to interactively plan the FOV in the healthy volunteers. Interactive planning mode offered the possibility for continuous MRI image acquisitions, while the acquisition plane was adjusted to each subject’s cardiac anatomy. In this study, we used interactive planning to align the image acquisition plane along the cardiac short axis.

### 2.2 Data collection

The following three paragraphs describe the adopted sequences for: 1) gold-standard T_1_ and T_2_ measurements (as reference for the phantom experiments); 2) clinically used T_1_ measurements (modified Look-Locker inversion recovery: MOLLI); 3) clinically used T_2_ measurements (gradient spin-echo: GraSE).

Reference gold-standard T_1_ and T_2_ measurements were performed on the MR-simulator to attain ground-truth values acquired independently in controlled settings. For gold-standard T_1_ mapping, we used an inversion recovery spin-echo sequence (TR/TE = 8000/8.7 ms, FA = 90°, FOV = 256×140 mm^2^, voxel size = 1×3×5 mm^3^) with 8 inversion times (TI = [100, 200, 400, 700, 1100, 1600, 2200, 2900] ms). For gold-standard T_2_ mapping, we used a single-echo spin-echo sequence (TR/TE  = 8000/8.7 ms, FA  = 90°, FOV  = 250×140 mm^2^, voxel size  = 1×3×5 mm^3^) with 8 echo times (TE = [18.7, 28.7, 48.7, 78.7, 118.7, 168.7, 228.7, 298.7] ms). The scan time for each gold-standard measurement was 50 min.

For T_1_ mapping, we used the multi 2D (M2D) balanced steady-state free precession (SSFP) MOLLI (5(3)3) sequence (TR/TE = 2.7/1.3 ms, FA = 35°, FOV = 350×193 mm^2^, voxel size = 2×2×10 mm^3^, SENSE = 2), clinically available at our department. This sequence, which is robust for heart rates exceeding 60 bpm [27], was utilized with cardiac triggering and breath-holds of 15 s for a single slice. The inversion times were automatically calculated by the scanner software based on the heart frequency of the volunteer. For cardiac triggering, the peripheral pulse signal detected by the PPU was used. This sequence was acquired twice on the MR-simulator and the MR-linac both for the phantom and the healthy volunteers (along the short axis plane of the heart). During the phantom measurements, a researcher was present in the scanner room wearing the PPU to provide the software with the peripheral pulse signal to ensure comparable scan settings with respect to *in vivo* measurements.

For T_2_ mapping, we used the M2D black blood-prepared GraSE sequence, clinically available at our department (TR  = 1 heartbeat, 11 echos, TE_eff_ = 11 ms, FA  = 90°, FOV  = 305×502 mm^2^, voxel size  = 2×2×10 mm^3^, SENSE  = 2), with breath-holds of 25 s for a single slice and cardiac triggering based on the peripheral pulse signal detected by the PPU. Similar to T_1_, this sequence was acquired twice on the MR-simulator and the MR-linac both for the phantom and the healthy volunteers (along the short axis plane of the heart). Again, a researcher was present in the scanner room during the phantom acquisitions wearing the PPU to ensure comparable scan settings with respect to *in vivo* measurements.

Note that the T_1_ and T_2_ sequences run on the MR-linac and MR simulator were the same to enable direct and unbiased comparisons. We did not modify/optimize the sequences between scanners as modifications in sequence parameters may lead to alterations of measured T_1_/T_2_ values as shown by Kellman et al. [27].

### 2.3 Data processing

Native T_1_ and T_2_ maps were directly reconstructed by the vendor software after acquisition on the scanner and saved as DICOM files. Using in-house developed software (clinically used for contouring in our radiotherapy department) [28], regions-of-interest (ROIs) were manually drawn for calculating the mean and standard deviation of the T_1_ and T_2_ relaxation times. In the phantom gel samples, a circular shaped ROI was used in the transversal plane. In the volunteer data, an ROI in the midseptal wall was used.

From the phantom experiments, the obtained T_1_ and T_2_ relaxation times using the clinical sequences were compared with the measured values using the gold-standard measurements by calculating the percentage error (PE) via the following equation:

(1) $$\mathrm{PE}=\frac{T_{x}-T_{x,\mathrm{ref}}}{T_{x,\mathrm{ref}}}\times 100\%,$$

where $T_{x,\mathrm{ref}}$ is the measured reference, ground-truth T_1_ or T_2_ value and $T_{x}$ is the measured T_1_ or T_2_ value from the clinical mapping sequence. A negative/positive PE indicates underestimated/overestimated relaxation time with respect to the relaxation times measured with the gold-standard sequences.

## 3 Results

### 3.1 Phantom measurements

**Fig. 1:**
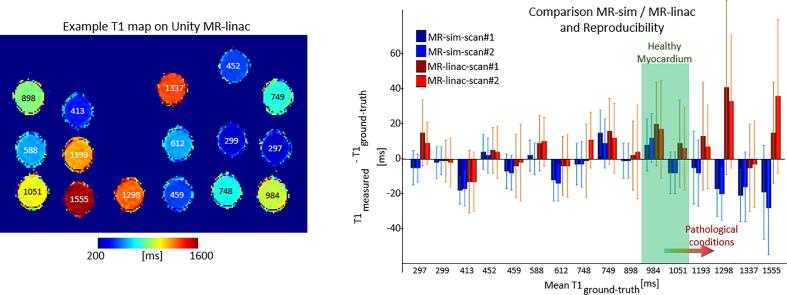
An example T_1_ map of the gel samples with the measured T_1_ values acquired on the MR-linac (left). The measured values with the clinically used sequence were subtracted by the reference, ground-truth measurement. The T_1_ value range for healthy myocardium is indicated with a green bar (right), while its variation for pathological conditions for RT applications (e.g., VT scar) is indicated by the red arrow [17].

**Fig. 2:**
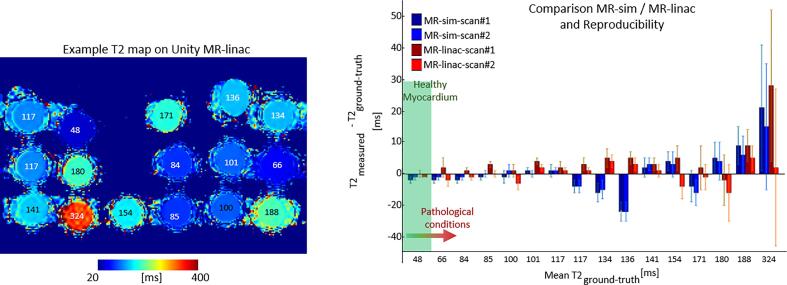
An example T_2_ map of the gel samples with the corresponding measured T_2_ relaxation times acquired on the MR-linac (left). The measured values with the clinically used sequence were subtracted by the reference, ground-truth measurement. The T_2_ value range for healthy myocardium is indicated with a green bar (right), while its variation for pathologic conditions is indicated by the red arrow [29], [30].

**Supplementary Fig. 2:**
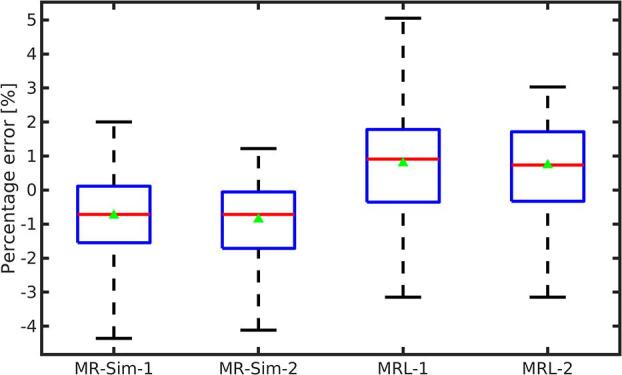


For the T_1_ measurements (Fig. 1), a maximum PE of −4.4% with respect to the corresponding ground-truth, gold-standard measurement was observed on the MR-simulator, while a maximum PE of −3.2% with respect to the corresponding ground-truth measurement was observed on the MR-linac. Within the relevant range for cardiac tissue, the maximum PE was lower: +1.2% on the MR-simulator, +2.0% on the MR-linac. For T_2_ measurements (Fig. 2), a maximum PE of −8.8% with respect to the corresponding ground-truth, gold-standard measurement was observed on the MR-simulator, while a maximum PE of  +8.6% with respect to the corresponding ground-truth measurement was observed on the MR-linac. Also for T_2_, within the relevant range for cardiac tissue the maximum PE was lower: −4.2% on the MR-simulator, −2.5% on the MR-linac. Box plots of the percentage errors for each separate measurement (two on both systems) are shown in the Supplementary Figs. 2 and 3.

### 3.2 In-vivo measurements

**Fig. 3:**
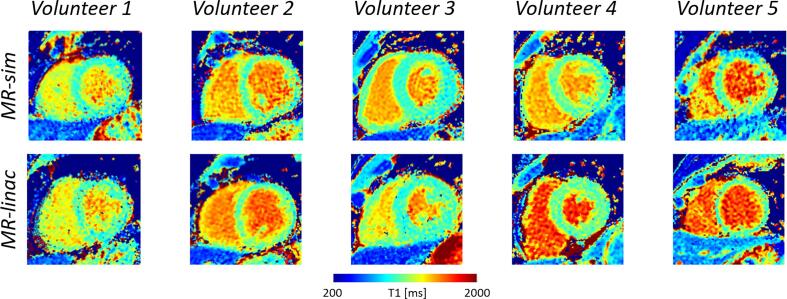
The acquired cardiac T_1_ maps of the healthy volunteers using the MR-simulator (top row) and MR-linac (bottom row) are shown in the short axis view at the mid-ventricular level.

**Fig. 4:**
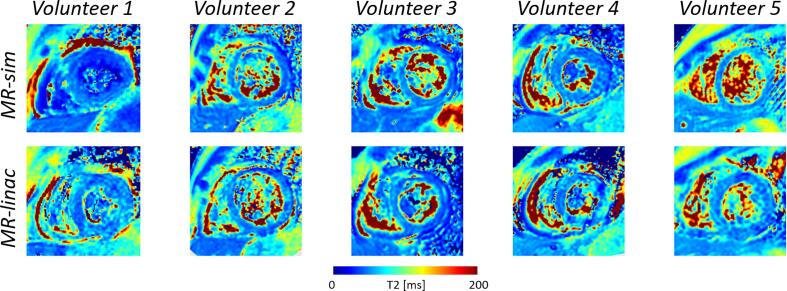
The acquired cardiac T_2_ maps of the healthy volunteers using the MR-simulator (top row) and MR-linac (bottom row) are shown in the short axis view at the mid-ventricular level.

**Fig. 5:**
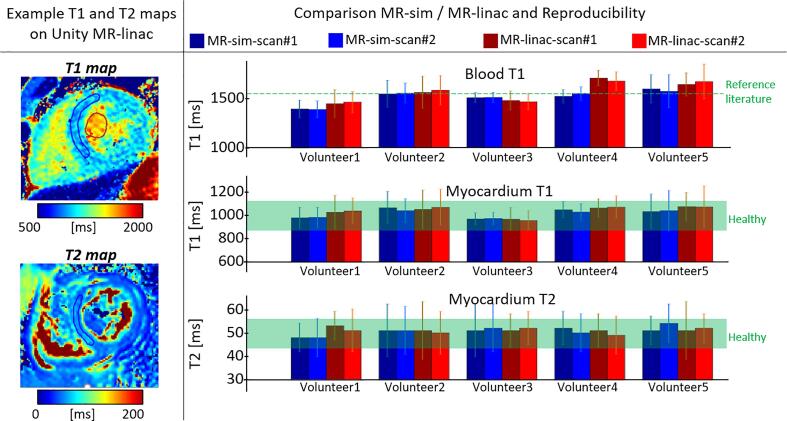
Example cardiac T_1_ and T_2_ maps are shown in the short axis orientation (left). The relaxation times in the corresponding ROI are compared (mean ± standard deviation). The corresponding value ranges for healthy myocardium and blood reported in literature are indicated with the green horizontal bar (right) [17], [29], [30].

The in vivo T_1_/T_2_ reconstructions showed comparable image quality between volunteers and also between MRI systems (Fig. 3 and Fig. 4). The absolute T_1_ and T_2_ relaxation times comparison of all scans in healthy volunteers is shown in Fig. 5 together with the corresponding ROI in the mid-septal wall and blood pool (for T_1_ only). The observed mean and standard deviation values show good intra/inter subject and intra/inter scanner agreement. The measured values also agreed with reported literature values on healthy subjects, as shown in the Fig. 5.

## 4 Discussion

To our knowledge, this is the first study in which cardiac-synchronized quantitative T_1_ and T_2_ MRI was performed on an MR-linac. Imaging hardware for cardiac synchronized MRI was successfully installed and used for in vivo cardiac T_1_ and T_2_ mapping on Unity in research mode. Phantom and in vivo measurements on healthy volunteers demonstrated that the acquired T_1_ and T_2_ quantitative maps on the MR-linac were in good agreement with the maps acquired on diagnostic MRI scanners for clinically used T_1_ and T_2_ MRI sequences. Based on these encouraging findings, we foresee that quantitative cardiac MRI on hybrid MR-linac systems might facilitate plan adaptation workflows for cardiac radioablation, or be used as imaging modality for (repeat) cardiotoxicity assessment.

Previous works showed the feasibility of quantitative MRI on 0.35 T [31], [32] and 1.5 T [26], [33], but not in the context of cardiac imaging. These studies demonstrated good agreement between their proposed qMRI methods and either gold-standard measurements or reference values on phantom. In particular, the work of Kooreman et al. investigated quantitative T_1_ and T_2_ mapping sequences on Unity MR-linac systems in detail [26]. However, a comparison with gold-standard measurements and measurements on diagnostic MRI systems were not performed.

In this study, phantom T_1_ and T_2_ measurements were compared against independently acquired gold-standard measurements providing ground-truth T_1_ and T_2_ values. These independent measurements were used as reference instead of the phantom vendor-provided values to reduce the potentially arising uncertainties in relaxation times due to, for example, room temperature, phantom gel stability and uncertainties in the vendor’s reference values (reported to be ± 3%). The good intra/inter scanner agreement between T_1_ and T_2_ measurements and their good agreement with gold-standard measurements demonstrate the robustness and reproducibility of the clinical sequences on Unity.

For the in vivo T_1_ and T_2_ maps, we observed comparable image quality and quantitative results between measurements and systems. The mean T_1_ values of the blood and myocardium across subjects were 1504 ± 70 ms and 1012 ± 34 ms, respectively, on the MR-simulator, and 1558 ± 99 ms and 1034 ± 42 ms, respectively, on the MR-linac. These values are in line with literature [17]. The mean T_2_ values of the myocardium were 51 ± 2 ms (measured on MR-simulator) and 51 ± 1 ms (measured on MR-linac), which were also in agreement with literature [29], [30]. This demonstrated the robustness and reproducibility of the clinically used T_1_ and T_2_ sequences. Additionally, the good inter-scanner agreement demonstrated in this study will be extremely important when target identification (e.g., myocardial scar) on MR-linac is based on quantitative values. A comparison (both on phantoms and in vivo) between MR-simulator and MR-linac measurements should be repeated for every MR-linac system used to acquire T_1_ and T_2_ sequences in order to ensure correct and reproducible quantification of cardiac tissue relaxation times across systems.

Quantitative T_1_ and T_2_ measurements are susceptible to environmental factors (e.g., temperature), but could also be susceptible to changes in imaging hardware. The MR-simulator is a diagnostic MRI scanner optimized for radiotherapy simulations in the treatment position. The hardware of the MRI system within the MR-linac is highly modified (e.g., split gradient coils) due to the addition of the linear accelerator, leading to considerable differences that may affect image acquisitions. As example, the gradient strength and slew rate of the diagnostic MR-simulator is 45 mT/m and 200 T/m/s versus 15 mT/m and 65 T/m/s on the 1.5 T Elekta Unity MR-linac [34]. In addition, the number of channels within the receive coils is different between systems: 8 on the MR-linac versus 32 on the MR-simulator. Despite these hardware differences, the selected sequences yielded highly comparable T_1_ and T_2_ maps.

Importantly, the adopted clinical T_1_ and T_2_ mapping sequences required cardiac synchronization, which was not a standard feature on the MR-linac. Cardiac synchronization for CMR imaging protocol would be ideally performed by continuously acquiring an electrocardiogram (ECG) signal with multiple electrodes attached on the skin. During this study, the preference for a PPU was deliberate to simplify volunteer scanning setup. The PPU triggers the image acquisition based on the peripheral pulse signal measured at the fingertip. The PPU signal is therefore inherently delayed with respect to the heart beat (R-R peaks). While this delay slightly varies per subject, the commonly used delay of 250 ms was also deployed in this study. For subjects with a fast heartbeat (>100 bpm), the PPU delay could lead to situations in which image quality might be degraded by cardiac motion-induced artifacts, since the MR image acquisition would not be fully restricted to the quiescent phase of the R-R interval (end diastole).

Respiratory motion mitigation was also required to minimize respiratory motion-induced artifacts. An air cushion (as shown in Supplementary Fig. 1 attached to the PPU device) was strapped down with a belt to measure a respiration signal. Based on this signal, the volunteers were asked to hold their breath in end-exhale. The duration for a single breath-hold was about 15 s for T_1_ mapping and 25 s for T_2_ mapping, which was subject to variations related to the heart rate of the volunteer. In radiotherapy, prolonged and repetitive breath-holds may be challenging for patients in a poor condition. Practical solutions could include longer recovery times for the patient between breath-holds, or compromises in the sequence parametrization to accelerate image acquisition (e.g. compared to the adopted one, a smaller in-plane FOV of 350×350 mm^2^ would lead to breath-holds of 17 s, but about 20% reduction in relative SNR). In a research setting, free-breathing T_1_ and T_2_ mapping sequences are being explored [35], [36].

Specifically for VT patients and occasionally for cancer patients, a cardiac implanted electronic device (CIED) is present to either maintain a sinus rhythm or to receive an ICD shock when a VT episode occurs. CIEDs were historically considered a contraindication for an MRI examination, but in recent years updated guidelines outlined the safe use of MRI in patients with CIEDs [37]. Safety considerations include the reduction of the maximum gradient strength, gradient slew rate, and subject specific absorption rate. Clinical T_1_ and T_2_ sequences used in this study already complied with the safety recommendations for patients with CIED. Clearly, the presence of a CIED may also affect the quality and usability of the acquired MRI images [38]. The impact of these artifacts on the image quality should therefore be assessed for each patient prior to treatment.

Additionally, parallel to tissue/scar characterization and cardiotoxicity assessment via T_1_/T_2_ CMR imaging, cardio-respiratory motion management is another key aspect for STAR treatments. Cine MRI techniques can be used for cardio-respiratory motion assessment as shown recently in [39], where cardiac motion can be estimated in a 2D plane on the MR-simulator and MR-linac. This information can be used to better define a planning target volume (PTV) that accounts for cardio-respiratory motion. For motion management during treatment, we then foresee three possible options: 1) passive management approach using a large PTV, with adapted margins to account for cardio-respiratory motion; 2) active approach using gating on the same cardiac phase used for anatomical/quantitative MRI using cardiac synchronization devices (ECG/PPU); 3) active approach using continuous tracking of cardiac motion (e.g., by using Gaussian processing [40]) with the multi leaf collimator (MLC) system. However, further research is required to allow adoption of these novel methodologies for guidance of STAR treatments.

Ultimately, cardiac-synchronized T_1_ and T_2_ qMRI was enabled on a 1.5 T MR-linac system. The accuracy and reproducibility of cardiac-synchronized T_1_ and T_2_ mapping was shown in phantom and *in vivo* experiments. By enabling the cardiac-synchronized qMRI feature, novel plan adaptation workflows and cardiotoxicity assessments might be facilitated.

## Declaration of Competing Interest

The authors declare the following financial interests/personal relationships which may be considered as potential competing interests: Eveline Alberts is an employee of Philips Healthcare.

## Figures and Tables

**Supplementary Fig. 3:**