# Supplementary material for: Feasibility of cardiac-synchronized quantitative T1 and T2 mapping on a hybrid 1.5 Tesla magnetic resonance imaging and linear accelerator system
Source: Phys Imaging Radiat Oncol. 2022 Mar 9;21:153–9. doi: 10.1016/j.phro.2022.02.017 (PMC8917300; doi:10.1016/j.phro.2022.02.017)
Supplement: Supplementary data 1 [file mmc1.docx]

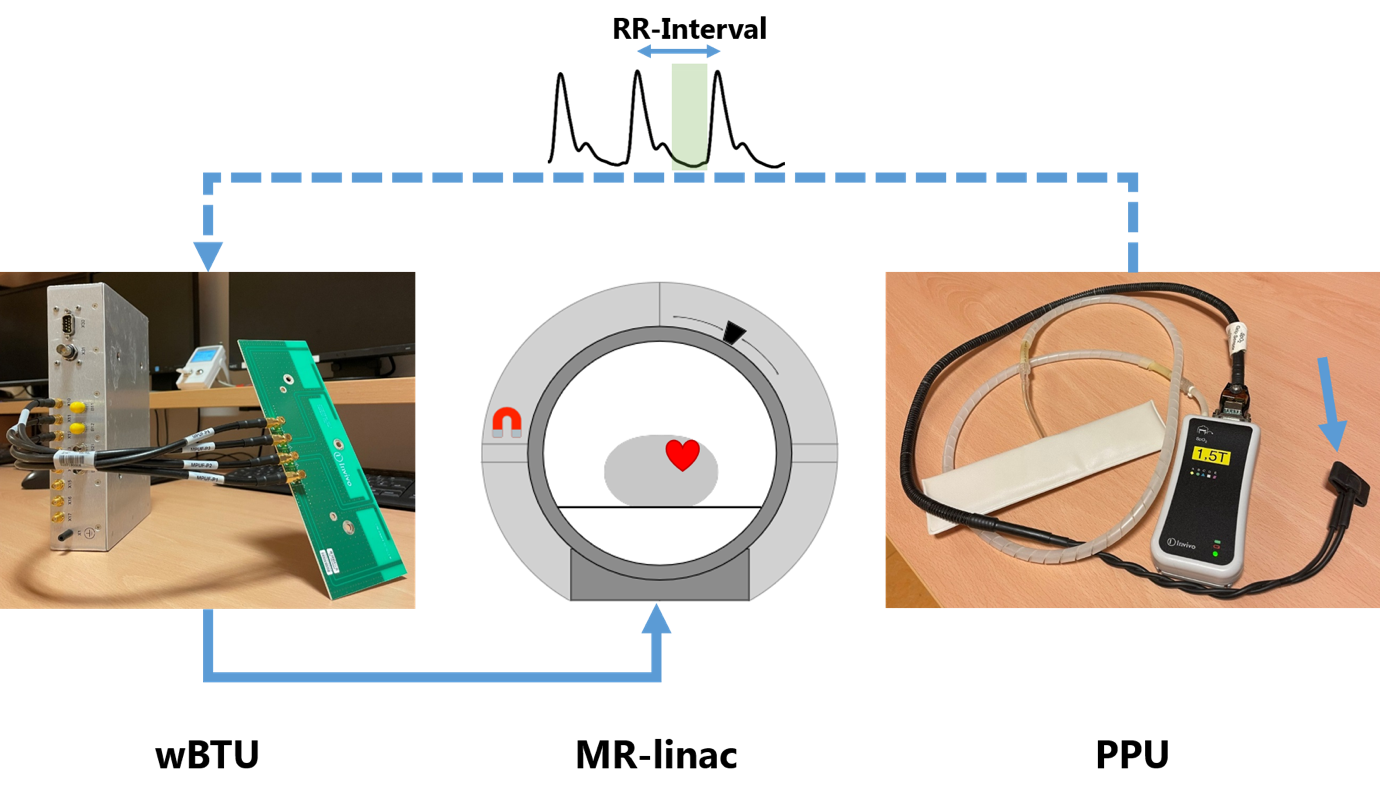


Supplementary Figure 1: A wireless peripheral pulse-oximeter unit (PPU) device measures a pulse signal from the subject’s fingertip (blue arrow) to detect its heart rate and is continuously streamed to the wireless basic triggering unit (wBTU). The wBTU has a wired connection with the MR-linac to trigger the MRI scan based on the received latest peripheral pulse signal for imaging in a pre-defined acquisition window (green bar in RR-interval).
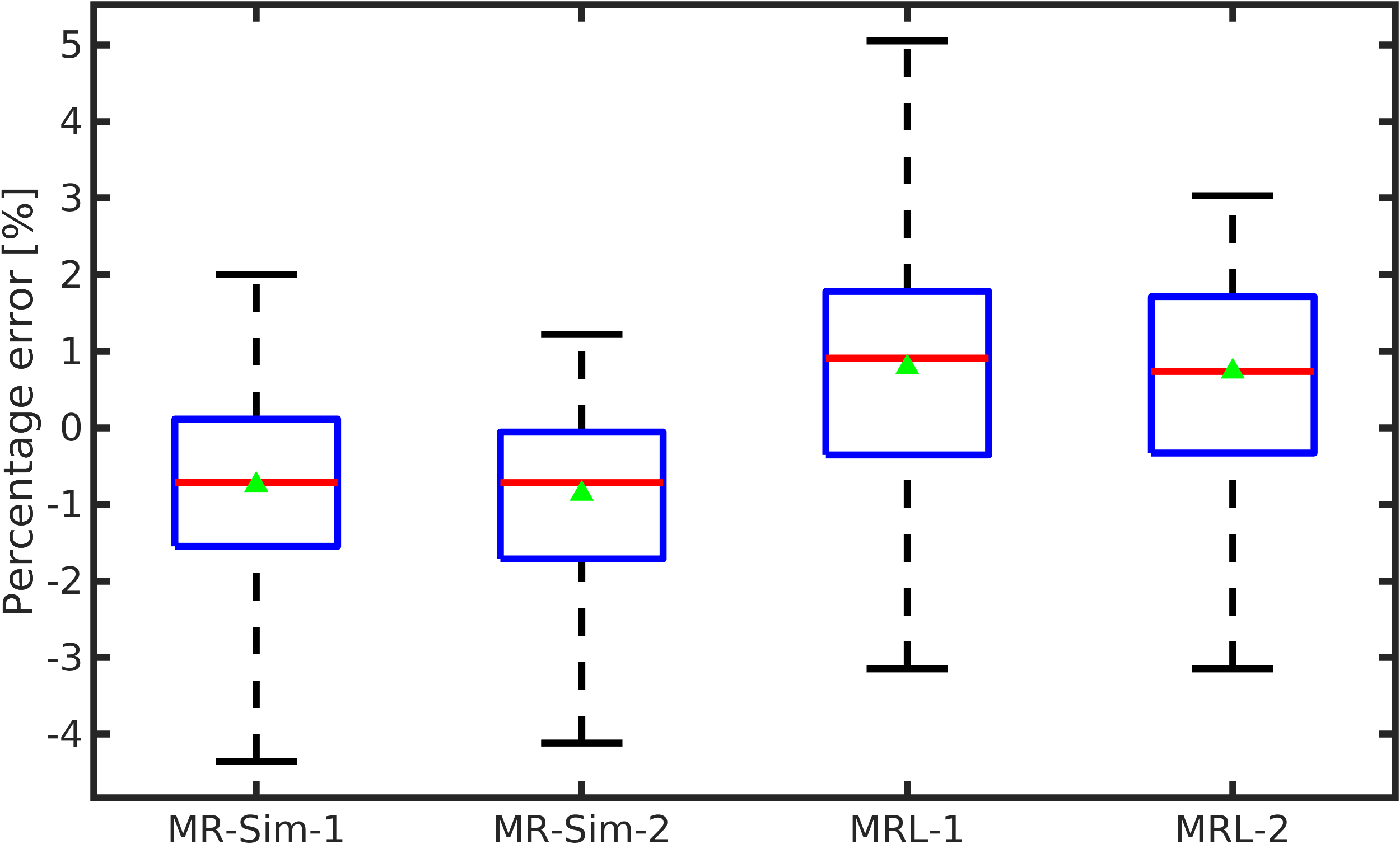


Supplementary Figure 2: Box plots of the percentage error with respect to the ground-truth measurement for each acquired clinical T_1_ map of the phantom gel samples are shown, in which MR-Sim-1 and MR-Sim-2 indicate measurements done on the MR-simulator and MRL-1 and MRL-2 indicate measurements on the MR-linac. The box plot shows the minimum, maximum, median, first quartile and third quartiles of the obtained percentage errors. The mean percentage error is indicated by the green triangular marker.


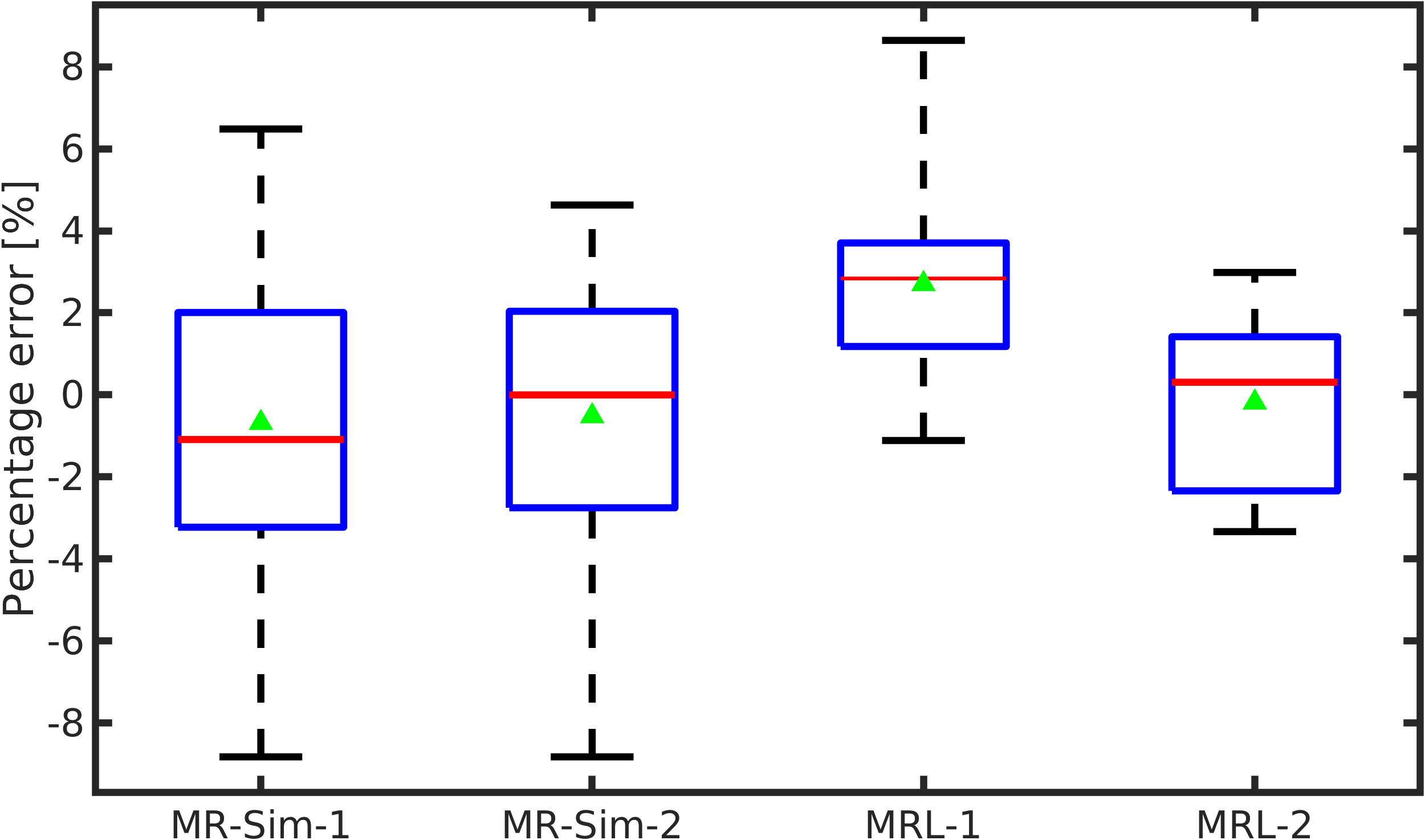


Supplementary Figure 3: Box plots of the percentage error with respect to the ground-truth measurement for each acquired clinical T_2_ map of the phantom gel samples are shown, in which MR-Sim-1 and MR-Sim-2 indicate measurements done on the MR-simulator and MRL-1 and MRL-2 indicate measurements on the MR-linac. The box plot shows the minimum, maximum, median, first quartile and third quartiles of the obtained percentage errors. The mean percentage error is indicated by the green triangular marker.
